# Genetics of SCID

**DOI:** 10.1186/1824-7288-36-76

**Published:** 2010-11-15

**Authors:** Fausto Cossu

**Affiliations:** 1 Pediatric HSCT Unit, 2^ Pediatric Clinic of University, Ospedale Microcitemico, Via Jenner s/n, 09121 Cagliari, Sardinia, Italy

## Abstract

Human SCID (Severe Combined Immunodeficiency) is a prenatal disorder of T lymphocyte development, that depends on the expression of numerous genes. The knowledge of the genetic basis of SCID is essential for diagnosis (e.g., clinical phenotype, lymphocyte profile) and treatment (e.g., use and type of pre-hematopoietic stem cell transplant conditioning).

Over the last years novel genetic defects causing SCID have been discovered, and the molecular and immunological mechanisms of SCID have been better characterized. Distinct forms of SCID show both common and peculiar (e.g., absence or presence of nonimmunological features) aspects, and they are currently classified into six groups according to prevalent pathophysiological mechanisms: impaired cytokine-mediated signaling; pre-T cell receptor defects; increased lymphocyte apoptosis; defects in thymus embryogenesis; impaired calcium flux; other mechanisms.

This review is the updated, extended and largely modified translation of the article "Cossu F: **Le basi genetiche delle SCID**", originally published in Italian language in the journal "*Prospettive in Pediatria*" 2009, **156:**228-238.

## Introduction

The initial clinical manifestations of SCID (Severe Combined Immunodeficiency), a heterogeneous group of genetic defects with an overall incidence of about 1 in 40,000 to 75,000 newborns [1-3], are most frequently observed in the first few months of life and the median age at diagnosis is 4-7 months. However, *human SCID is a prenatal disorder of T lymphocyte development, already present at birth even if clinically silent in most affected newborns*.

**Figure 1:**
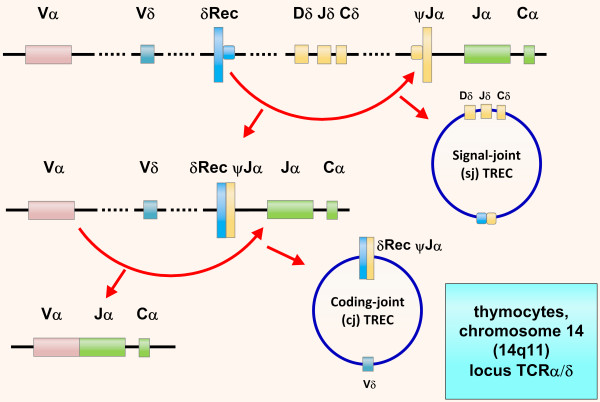
**T cell Receptor Excision Circles (TRECs)**. TRECs are episomal DNA circles produced in thymocytes by excisional rearrangements of T cell receptor (TCR) genes; they are stable, not duplicated during mitosis, diluted out with each cell division, and therefore higher in thymocytes, recent thymic emigrants (RTEs) and naïve T cells. Quantitative polymerase chain reaction (PCR) of coding-joint (cj) δRec ψJα TREC, produced at TCRα/δ locus within chromosome 14 (14q11) by > 70% of developing human α:β T cells, counts in the peripheral blood naïve α:β T lymphocytes recently dismetted by thymus: in newborn, values < 25 TRECs/μL indicate SCID.

On January 1, 2008, Wisconsin (USA) became the first state in the world to screen all newborns for SCID through a method based on measurement of T cell receptor excision circles (TRECs) by polymerase chain reaction (PCR), using DNA extracted from newborn dried blood spots (Guthrie cards); TRECs are by-products generated during normal T cell maturation (Figure 1) and are consistently absent or present in very low numbers in newborns with SCID [4]. Recently an infant with SCID has been identified by newborn screening in Massachusetts [5], and the U.S. Department of Health and Human Services recommended the addition of SCID to the uniform screening panel for all newborns [6].

**Figure 2:**
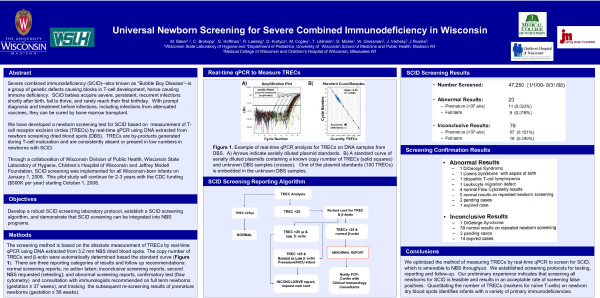
**Wisconsin Newborn SCID screening poster**. Reproduced with kind permission of the WI State Laboratory of Hygiene, *http://www.slh.wisc.edu/posters/Baker102808.pdf*.

Wisconsin SCID screening poster (Figure 2) describes the fundamental features of SCID: children with SCID do not produce T lymphocytes (or, however, functional T lymphocytes), acquire multiple, persistent and severe viral, bacterial and fungal infections shortly after birth, fail to thrive, and rarely reach their first birthday; SCID is a pediatric emergency [7]: with prompt diagnosis and treatment and before acquiring an infection, including infections from "live" vaccines (e.g., Bacille Calmette-Guérin, and recently rotavirus) [8,9], essentially every baby with SCID could be cured by *hematopoietic stem cell transplantation *(**HSCT**) or *gene therapy *(**GT**).

It is very useful to remember *other general aspects of SCID*:

• *Most newborns with SCID appear normal and healthy at birth*; slight cutaneous signs similar to GvHD (Graft versus Host Disease) from engraftment of transplacentally derived maternal T lymphocytes are sometimes present. Instead, low birth length and weight, microcephaly, dysmorphic facies, metaphyseal chondrodysplasia or other skeletal abnormalities, alopecia, congenital heart disease, etc. are nonimmunological manifestations of the less frequent forms of SCID in which cell types and organs other than lymphocytes and lymphoid organs are also affected by their genetic mutations (Table 1).

**Table 1:** Classification of SCID.

Prevalent mechanisms/Disease	T/B/NK	Gene	Locus	Heredity	Protein ^§§^	Nonimmunological manifestations
**Impaired cytokine-mediated signaling**						

Common γ chain defect	T^-^B^+^NK^-^	*IL2RG*	Xq13.1	XL	Common γ chain	

JAK3 defect	T^-^B^+^NK^-^	*JAK3*	19p13.1	AR	Janus kinase 3	

IL-7Rα chain defect	T^-^B^+^NK^+^	*IL7RA*	5p13	AR	IL-7 and TSLP receptor α chain	

**Defects of the pre-T cell receptor**						

***Defects in V(D)J recombination***						

RAG1 defect	T^-^B^-^NK^+^	*RAG1*	11p13	AR	RAG1	

RAG2 defect	T^-^B^-^NK^+^	*RAG2*	11p13	AR	RAG2	

Artemis defect	T^-^B^-^NK^+^	*DCLRE1C*	10p13	AR	Artemis	radiosensitivity

DNA-PKcs defect	T^-^B^-^NK^+^	*PRKDC*	8q11.21	AR	DNA-PKcs	radiosensitivity

DNA ligasi IV defect	T^-^B^-^NK^+^	*LIG4*	13q33.3	AR	DNA ligasi IV	radiosensitivity, dysmorphic facies, microcephaly, growth retardation, psychomotor delay

Cernunnos/XLF defect	T^-^B^-^NK^+^	*NHEJ1*	2q35	AR	Cernunnos/XLF	radiosensitivity, dysmorphic facies, microcephaly, growth retardation, psychomotor delay

***Impaired signaling through the pre-T cell receptor***						

CD3δ defect	T^-^B^+^NK^+^	*CD3D*	11q23	AR	CD3δ	

CD3ε defect	T^-^B^+^NK^+^	*CD3E*	11q23	AR	CD3ε	

CD3ζ defect	T^-^B^+^NK^+^	*CD3Z*	1q24.2	AR	CD3ζ	

CD3γ defect	T^-^B^+^NK^+^	*CD3G*	11q23	AR	CD3γ	

CD45	T^-^B^+^NK^-/+^	*PTPRC*	1q31.3	AR	CD45 (LCA)	

ZAP-70 defect	T^+^B^+^NK^+^	*ZAP70*	2q11.2	AR	ZAP-70	
	CD4^+ ^CD8^-^					

p56lck defect	T^-^B^+^NK^+^	*LCK*	1p35.1	AR	p56lck	

**Increased lymphocyte apoptosis**						

Reticular dysgenesis	T^-^B^-^NK^-^	*AK2*	1p34	AR	Adenylate kinase 2	aleukocytosis, sensorineural deafness

ADA-SCID	T^-^B^-^NK^-^	*ADA*	20q13.11	AR	Adenosine deaminase	costochondral and skeletal alterations, neonatal hepatitis, sensorineural deafness, neurological problems

PNP-SCID	T^-^B^-^NK^-^	*PNP*	14q11.2	AR	Purine nucleoside phosphorylase	neurological problems

**Defects in thymus embryogenesis**						

Nude/SCID Syndrome	T^-^B^+^NK^+^	*WHN*	17q11.2	AR	FOXN1	alopecia; embryonic neural tube defects

***Complete DiGeorge Anomaly***						

DiGeorge Syndrome (del22q11.2)	T^-^B^+^NK^+^	*> 35 genes*	22q11.2	AD	TBX1, and others	dysmorphic facies, congenital heart disease and other malformations, neonatal hypocalcemia by absence of parathyroid glands

CHARGE	T^-^B^+^NK^+^	*CHD7*	8q12.1	AD	CHD-7	CHARGE association (coloboma, heart defects, atresia choanae, retardated growth and development, genital hypoplasia, ear anomalies/deafness)

Diabetic mother embryopathy	T^-^B^+^NK^+^					congenital heart disease, gut and kidney malformations, neural tube defects, sacral agenesis, holoprosencephaly, neonatal hypoglycemia

**Impaired calcium flux**						

ORAI1 defect	T^+^B^+^NK^+^	*ORAI1*	12q24	AR	ORAI1	myopathy, ectodermal dysplasia

STIM1 defect	T^+^B^+^NK^+^	*STIM1*	11p15.5	AR	STIM1	myopathy, ectodermal dysplasia

**Other mechanisms**						

Coronin-1A defect	T^-^B^+^NK^+^	*CORO1A*	16p11.2	AR	Coronin-1A	

MHC Class II defect	T^+^B^+^NK^+^	*CIITA*	16p13.13	AR	CIITA	
	CD4^- ^CD8^+^	*RFXANK*	19p13.11	AR	RFXANK	
		*RFX5*	1q21.2	AR	RFX5	
		*RFXAP*	13q13.3	AR	RFXAP	

CHH (Cartilage hair hypoplasia)	T^-^B^+^NK^+^	*RMRP*	9p13.3	AR	**^§§ ^**RNA of RNase MRP complex	short-limbed dwarfism, light-colored hypoplastic hair

Hoyeraal-Hreidarsson Syndrome (HHS)	T^+^B^-^NK^-^	*DKC1*	Xq28	XL	Dyskerin	cerebellar hypoplasia, microcephaly, growth retardation, bone marrow failure, hypoplastic hair
		*TERT*	5p15.33	AR	TERT	
		*TINF2*	14q12	AD	TIN2	
		*DCLRE1B*	1p13.2	AD	Apollo	

Hereditary folate malabsorption (HFM)	T^+^B^+^NK^+^	*SLC46A1*	17q11.2	AR	PCFT	megaloblastic anemia, seizures, risk of severe neurodevelopmental defects

• As noted above, even if most newborns with SCID appear normal at birth, *SCID is always a prenatal disorder of the development of T lymphocytes and it is already present at birth*. In fact, the newborn screening through TRECs does neither measure enzyme activity nor search for mutations: it only counts normal naïve T lymphocytes, already absent or markedly reduced. Note that unlike mice (in which, contrary to humans, neonatal thymectomy causes SCID) normal development of the human immune system starts very early and it is notably advanced before birth: in absence of SCID, in human embryos (since 9-10 weeks of age) there is intensive thymic T lymphopoiesis; and human in utero exposure to foreign antigens does activate immunological response and does not produce tolerance, apart that toward noninherited maternal alloantigens (tolerance mediated by specific regulators T lymphocytes CD4^+^CD25^high^FoxP3^+ ^T_Reg_, that represent 15-20% of CD4^+ ^T lymphocytes in the peripheral lymphoid organs of the human fetus) [10]. Therefore, in utero HSCT had success (also if usually partial: graft of T cells but not B cells) in human SCID fetuses, but failed completely - 0% successful (!!) with moreover 24% "procedure-related death" - in 17/17 fetuses with hemoglobinopathies (thalassemia, sickle cell disease) and normal immune system [11].

• The development and function of *T lymphocytes *are severely compromised in all forms of SCID ("congenital severe T cell immunodeficiencies"); however, T lymphocytes, B lymphocytes and NK (natural killer) lymphocytes (note that *NK cells, unlike T and B lymphocytes, do not rearrange their germline DNA to produce genes encoding antigen-specific receptors*) share progenitors for cell lineages, signaling pathways in development and function, and metabolic pathways. Therefore, *also B lymphocytes and/or NK cells are usually severely compromised in SCID*, and the distinct forms of SCID are characterized by different *combinations of T/B/NK counts*: **T^-^B^-^NK^-^, T^-^B^+^NK^-^, T^-^B^-^NK^+ ^, T^-^B^+^NK^+ ^**(^- ^means absence or severely reduced counts). Moreover, without normal CD4^+ ^T helper lymphocytes (T_H_1, T_H_2, T_Reg_, T_FH_, T_H_17, T_H_22, T_H_9) [12,13], B lymphocytes (in SCID *agammaglobulinemia *is the rule, with rare exceptions), macrophages and also eventual residual T lymphocytes cannot work even if present and "normal".

• In most SCID the *absence of T lymphocytes *causes marked *lymphopenia*, with often an absolute lymphocyte count (ALC) < 500 cells/μL. Note that in adults lymphopenia means ALC < 1,000/μL, but the normal lower limits are 2,000/μL in newborns and 4,000/μL in infants by 6 to 9 months of age; therefore, in the first few months of life any ALC < 2,500/μL is potentially pathogenic and may indicate SCID [14].

• However, *many infants with SCID have T cells*, showing slightly reduced, normal (**T^+ ^SCID**) or high (**T^++ ^SCID**) T cell counts: e.g., "functional" T^+^B^+^NK^+ ^SCID in the defects of calcium channels [15], T^+ ^(CD4^+^CD8^-^) B^+^NK^+ ^SCID in the defect of ZAP70 [16], T^+^B^-^NK^- ^SCID in the Hoyeraal-Hreidarsson syndrome [17].

**Figure 3:**
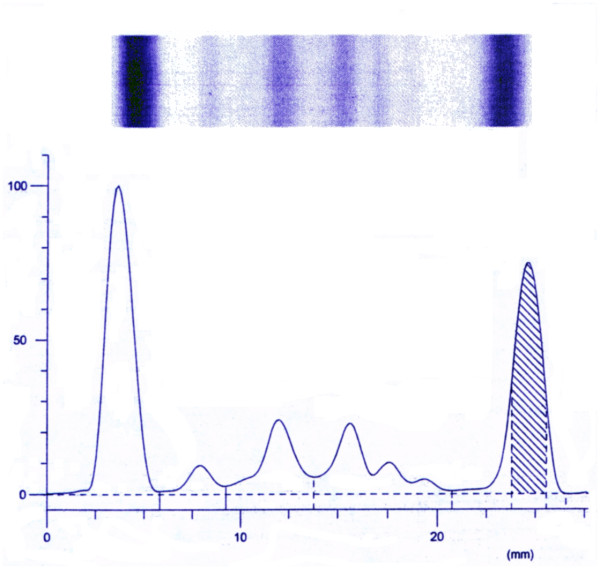
**Monoclonal IgG gammopathy in a Sardinian 4-month-old female infant**. AR T^-^B^-^NK^+^SCID, modified to T^++^B^-^NK^+ ^SCID by massive maternal T lymphocyte engraftment; ALC 16,740/μL, IgG 3,390 mg/dL; homozygous frameshift > nonsense mutation of *DCLRE1C *gene (Artemis defect).

But, T^+ ^or T^++ ^SCID are most frequently due to *abnormal and oligoclonal T cells *that modify counts from T^-^B^-^NK^- ^to T^+^B^-^NK^-^, from T^-^B^+^NK^- ^to T^+^B^+^NK^-^, from T^-^B^-^NK^+ ^to T^+^B^-^NK^+^, or from T^-^B^+^NK^+ ^to T^+^B^+^NK^+^. Such abnormal T lymphocytes (oligoclonal Vβ TCR families; very low naïve CD4^+^CD45RA^+ ^T cells; high memory CD4^+^CD45RO^+ ^T cells; high activated CD3DR^+ ^T cells; very low/absent in vitro mitogen-induced lymphocyte proliferation; and, very low/absent TRECs, that is very important regard to newborn SCID screening) are present in two main conditions:

**1) SCID with massive engraftment of transplacentally derived maternal T lymphocytes**: such maternal T lymphocytes are very useful for the diagnosis of SCID (HLA typing of infant's peripheral blood; maternal DNA in infant's peripheral blood), and may also cause various and unusual manifestations: skin and liver GvHD [18], autoimmune thrombocytopenia or pancytopenia (pre- or post-HSCT), rejection of HSCT from father or donors other than mother [19], monoclonal gammopathy because of clonal expansion of maternal or newborn B cells in absence of normal CD4^+^T_Reg _lymphocytes (Figure 3) [20], attenuated clinical SCID if fetus/mother HLA compatibility [21].

**Figure 4:**
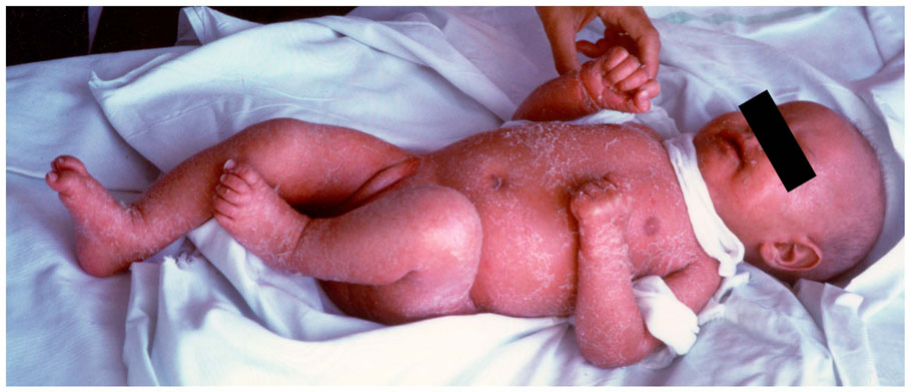
**Omenn Syndrome in a Sardinian 5-month-old female infant (absence of *RAG1-RAG2 *mutations, unidentified gene defect)**. "Leaky" mutations of practically all SCID genes (whose null mutations cause instead typical SCID) produce Omenn syndrome, in fact described in infants with defects of RAG1-RAG2, *DCLRE1C*-Artemis, ADA, DNA Ligasi IV, *RMRP*-CHH, common γc, IL7Rα, *WHN*-FOXN1, ZAP-70, and complete DiGeorge anomaly (DiGeorge Syndrome; CHARGE). In many infants with Omenn syndrome, that is clinically not leaky but very serious, genetic defect remains unidentified (several known, and probably also unknown, genes to be sequenced).

**2) Omenn Syndrome: **caused by hypomorphic mutations ("leaky SCID") not only of *rag1-rag2 *(recombination activating gene 1-2) [22] but also of *almost all other genes whose null mutations cause instead typical SCID *(Figure 4) [16,23-32]. Clinically, Omenn syndrome is not a "leaky" SCID and has a poor prognosis; it is an extremely serious T^+ ^or T^++ ^SCID with pathogenic *child's (not maternal) *autologous oligoclonal hyper-autoreactive CD4^+ ^T_H_2 lymphocytes, produced because of non-null mutations and then expanded by lack of central and peripheral immunological tolerance (respectively: thymic defect of thymocyte-dependent epithelial and dendritic cells and of AIRE, Autoimmune Regulator Element, expression; and defect of CD4^+^T_Reg _lymphocytes) [23,33-35]. Besides the overwhelming and life-threatening infections typical of SCID, Omenn infants present with aggressive tissue inflammation, very severe erythroderma (absent at birth), protein loss through the skin and the gut, unmanageable diarrhea, generalized edema, metabolic alterations, raised serum IgE, hypereosinophilia, alopecia and loss of eyebrows and eyelashes, enlarged lymph nodes and epatosplenomegaly; usually, signs and symptoms do not appear simultaneously and evolve with time [23].

• Therefore, mutations of the same gene can cause both typical SCID and Omenn syndrome but also various atypical clinical pictures and moreover slight immunological defects with later manifestations in adult age [36,37]. The *variability of the clinical phenotypes *is a common aspect of the genetic defects causing SCID [38], and it is observed also in siblings with the same mutation (interference of other genetic factors, environmental factors, etc.) [39,40]. In certain cases, clinical phenotypes are attenuated because of somatic mosaicism by spontaneous *genetic reversion *(correction of the mutation) in a somatic cellular line that then expands itself [41].

## Genotypes and clinical aspects of SCID

With the exception of the complete DiGeorge anomaly and some cases of the Hoyeraal-Hreidarsson syndrome (autosomal dominant, AD), all forms of SCID are autosomal recessive (AR-SCID) or X-linked recessive (XL-SCID) monogenic disorders.

An updated classification of SCID is based on underlying genetics and prevalent molecular pathogenetic mechanisms (Table 1).

### SCID caused by impaired cytokine-mediated signaling

#### Common gamma chain (γc) defect (OMIM, Online Mendelian Inheritance in Man, 300400)

**Figure 5:**
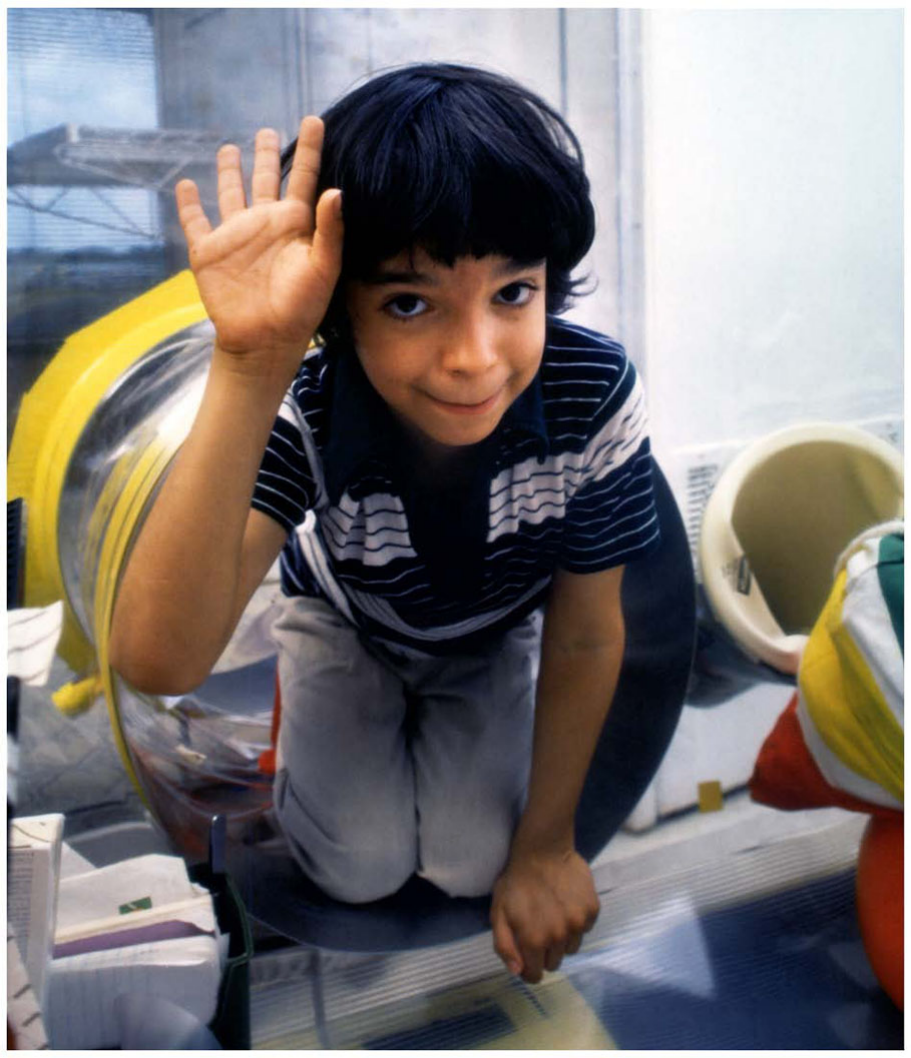
**David Vetter, the "Bubble Boy" (September 21, 1971 - February 22, 1984)**. David Vetter, photograph reproduced with kind permission of Prof. William T. Shearer, The David Center, Baylor College of Medicine, Texas Children's Hospital.

**Figure 6:**
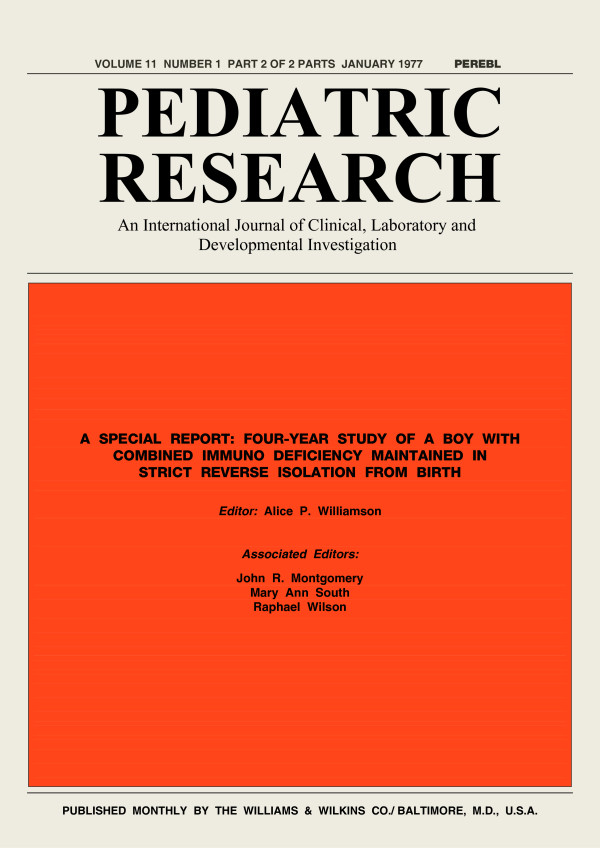
**Pediatric Research 1977, January**. In January 1977, a special issue of Pediatric Research (cover) reported about David Vetter.

Only male infants are affected by common gamma chain (γc) defect, XL T^-^B^+^NK^-^SCID caused by mutations of *IL2RG *gene, localized at Xq13.1 and encoding the γ chain (γc) of interleukin-2 (IL-2) receptor. David Vetter (Figure 5; Figure 6) was affected by this SCID (his mutation: *IL2RG *nonsense exon 7 C937A S308X, -62 aa. of 369 aa.) [42-45]; the "bubble boy paradox" (IL-2 deficient knockout mice and the rare IL-2 deficient human patients do not have SCID but instead severe defect of CD4^+ ^T_Reg _lymphocytes and autoimmunity) was resolved by the discovery that IL-2 receptor γ chain is shared (*common *γ chain) by the receptors of IL-4, IL-7, IL-9, IL-15, and IL-21; IL-7 and IL-15 are essential for the development of T lymphocytes and NK cells, respectively [46,47].

At least in the United States, this XL-SCID ("X-SCID") is the most frequent SCID; it is an exclusively immunological form except that also cutaneous keratinocytes express γc-dependent cytokine receptors and the associated JAK3 (see later), and these is necessary for the local innate immunity of keratinocytes against human papilloma virus (HPV); therefore, severe cutaneous HPV disease, including epidermodysplasia verruciformis, is a frequent late complications in common γc (and JAK3) SCID patients many years after successful HSCT or gene therapy [48].

In X-SCID fully HLA-matched sibling or family donor HSCT without any conditioning is successful in > 95%, however with frequent failure of donor B cell engraftment [49]. By contrast, history of gene therapy in X-SCID is very controversial [50].

#### JAK3 (Janus kinase 3) defect (OMIM 600802)

JAK3 defect is the AR T^-^B^+^NK^-^SCID equivalent of the common γc defect X-SCID, since that JAK3 joins to γc in the [IL-2,4,7,9,15,21] - JAK3 - STAT5 signaling pathway [47,51,52]. Most JAK3 SCID patients are compound heterozygotes for two different mutations [5,51].

#### IL-7Rα (IL-7 receptor alpha chain) defect (OMIM 608971)

IL-7Rα defect is an AR T^-^B^+^NK^+ ^SCID because IL-15 function (---> NK cells) is normal and by contrast functions of IL-7 and of TSLP (thymic stromal lymphopoietin), that share the α chain in their receptors, are compromised [47,53,54]. In humans, IL-7 produced by stromal cells of lymphoid tissues and by hepatocytes is the true "T cell growth development factor" [55], and so its dosage in Guthrie card eluate is a complementary (respect to TRECs test) method for newborn SCID screening: blood IL-7 is increased "by feedback" if low T lymphocytes, and newborn IL-7 levels > 15 pg/mL indicate T^- ^SCID [56]; note that IL-7 levels may be normal in T^+ ^or T^++ ^SCID (e.g., massive engraftment of maternal T lymphocytes, and Omenn syndrome; see before), and therefore TRECs test appears to be the best method for newborn SCID screening.

### SCID caused by pre-T cell receptor defects

The pre-T cell receptor (pre-TCR), formed by a TCRβ chain (rearranged *TCRβ *gene) and by the disulfide-linked invariant pre-TCRα chain (pTα chain; codified by the *PTCRA *gene, localized at 6p21.2, and really a very suspect candidate gene for SCID) has an essential role during T lymphopoiesis in thymic microambient at the stage of large pre-T cell [57]. The pre-TCR also transmits its signal through numerous other molecules common to the TCR (T cell receptor of mature T lymphocytes): CD3 complex (CD3γ, CD3δ, CD3ε, CD3ζ), protein-tyrosine kinases (e.g., Fyn, Lck, ZAP-70), protein-phosphotyrosine phosphatases (CD45, and others), etc. [58]

**Figure 7:**
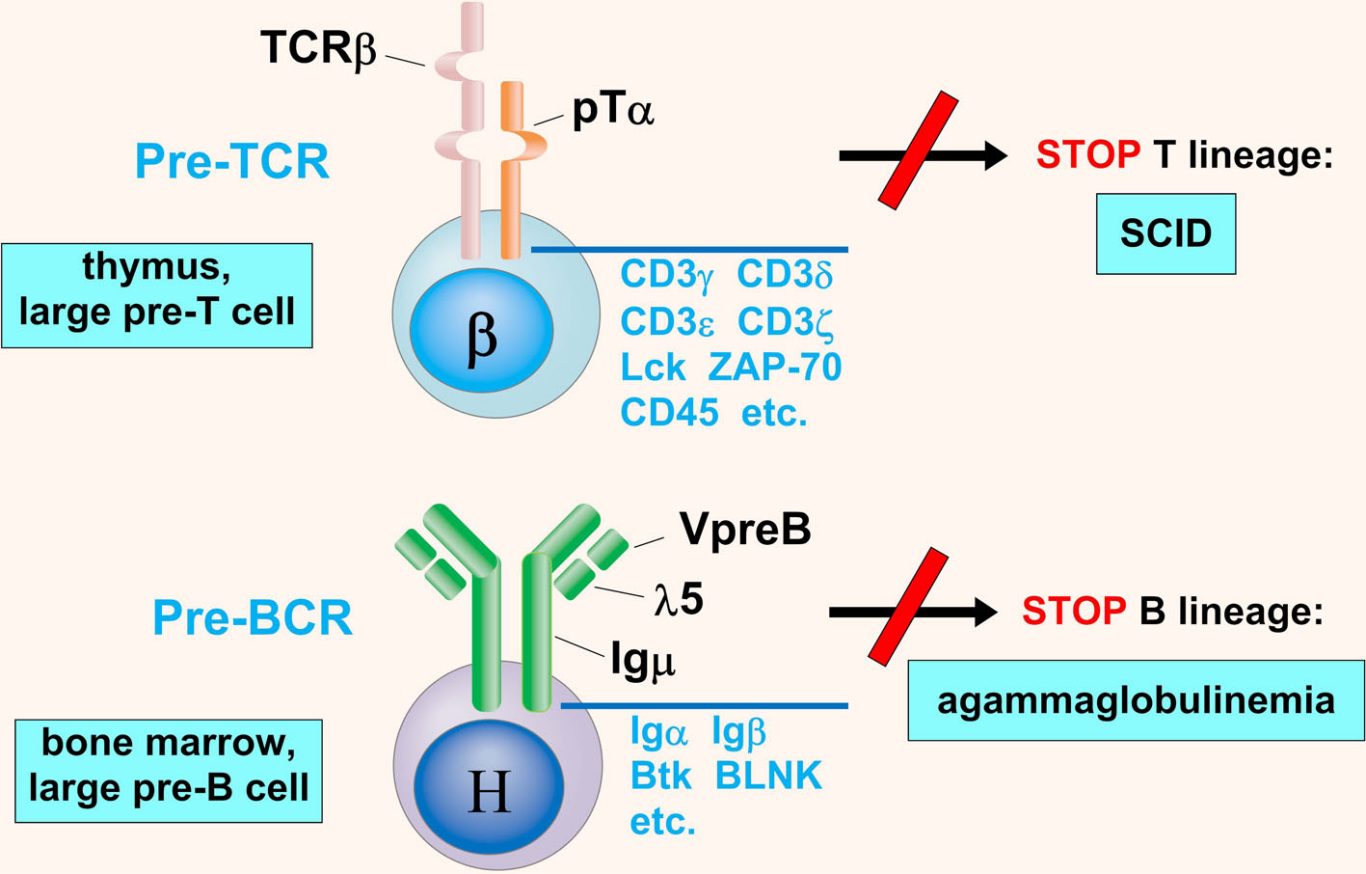
**Pre-TCR and pre-BCR**. Schematic drawing of pre-T cell receptor (pre-TCR; thymus, large pre-T cell with rearranged TCRβ gene) and pre-B cell receptor (pre-BCR; bone marrow, large pre-B cell with rearranged IgHμ gene). Defects of the pre-TCR, subdivided into defects of V(D)J recombination and defects of signaling through the pre-T cell receptor, cause arrest of the development of T lymphocytes at the stage of large pre-T cell and therefore SCID; defects of the pre-BCR cause arrest of the development of B lymphocytes at the stage of large pre-B cell and therefore agammaglobulinemia.

It is evident the similarity with the pre-B cell receptor (pre-BCR) of B lymphopoiesis: defects of the pre-BCR (Igμ, λ/VpreB, Igα, Igβ, Bruton tyrosine kinase BTK, B cell linker protein BLNK) cause arrest of the development of B lymphocytes at the stage of large pre-B cell and therefore *agammaglobulinemia *[59]; defects of the pre-TCR, subdivided into 1) *defects of V(D)J recombination *and 2) *defects of signaling through the pre-T cell receptor*, cause arrest of the development of T lymphocytes at the stage of large pre-T cell and therefore *SCID *(Figure 7).

#### 1) Defects of V(D)J recombination

The variable antigen-specific regions of the TCR β chain of pre-TCR, Igμ chain of pre-BCR and then TCR, BCR and Ig chains are encoded by the correspondent gene domains rearranged through the *V(D)J recombination of DNA *[60]. This recombination happens in *two steps: *1^st ^step *specific to T and B lymphocytes *(RAG1 and RAG2 "transposases", encoded by the "Recombination Activating Genes" *RAG1 *and *RAG2*); and 2^nd ^step due to the "Non-Homologous End-Joining" (NHEJ) proteins - Ku70/80, DNA-PKcs, Artemis, Cernunnos/XLF, DNA ligase IV, XRCC4 - pathway, that repairs double-strand breaks in DNA *in all living cells *[61].

Defects of all these molecules cause T^-^B^-^NK^+ ^SCID because, again, note that contrary to T and B lymphocytes NK cells do not rearrange their germline DNA to produce genes encoding antigen-specific receptors. In defects of 1^st ^step (RAG1, RAG2), abnormalities are limited to T and B lymphocytes; by contrast, defects of 2^nd ^step (NHEJ) affect all cells and cause problems similar to other syndromes with DNA repair defects: cellular *radiosensitivity*, extreme toxicity by pre-HSCT conditioning (especially by alkylating agents and, obviously, by irradiation), predisposition to neoplasia, and (but only in some forms: defect of DNA ligase IV, defect of Cernunnos/XLF) dysmorphic facies, microcephaly, psychomotor delay [62].

##### RAG1 or RAG2 defect (OMIM 601457)

RAG1 or RAG2 defect causes typical T^-^B^-^NK^+ ^SCID, Omenn syndrome, and also some rare peculiar forms: immunodeficiency with early onset of multivisceral and recurrent CMV infection, autoimmune cytopenia, restricted T cell repertoire with TCRαβ^+ ^lymphopenia and markedly expanded TCRγδ^+ ^T cells; immunodeficiency with extensive granulomatous disease involving the skin, mucous membranes and internal organs, and EBV-lymphoma; etc. [36,63]

In humans have been identified radiosensitive T^-^B^-^NK^+ ^SCID due to defects of *four NHEJ molecules*:

##### Artemis defect (OMIM 602450)

Artemis defect, due to mutations of the *DCLRE1C *(DNA cross-link repair protein 1C) gene, is present with various mutations in all world populations; once it was known as "SCIDA", that is SCID of Athabascan-speaking Native Americans (Apache, Navajo), in which because of a founder effect 1 person every 10 is heterozygote for the mutation exon 8 C576A Artemis Y192X and 1 in 2,000 newborn is affected [64].

##### DNA ligase IV defect (OMIM 606593)

DNA ligase IV defect causes the "Ligase IV syndrome" (phenotypically similar to the Nijmegen breakage syndrome, NBS) with possible typical SCID or Omenn syndrome, dysmorphic facies, microcephaly, growth retardation, psychomotor delay, skin anomalies, pancytopenia, and predisposition (also in heterozygotes) to leukemia and other neoplasia [65].

##### Cernunnos/XLF defect (OMIM 611291)

Cernunnos/XLF defect causes combined immunodeficiency (usually manifested later respect to typical SCID) with short stature, multiple dysmorphisms, microcephaly, psychomotor delay, bone marrow failure and myelodysplasia [66].

##### DNA-PKcs defect

Recently in a girl from consanguineous parents of Turkish origin, diagnosed with AR T^-^B^-^NK^+ ^SCID when she was 5-month-old, has been identified the first human case of DNA-PKcs (DNA-dependent Protein Kinase catalytic subunit) defect, due to a homozygous missense mutation (T9185C L3062R) of the coding gene *PRKDC *(OMIM 600899). It was known since many years that mutations of *PRKDC *(Protein kinase, DNA-activated, catalytic polypeptide) gene cause the naturally occurring SCID in mice, Arabian foals and Jack Russell terriers; so, DNA-PKcs defect has been long predicted in human SCID and finally found. Note that the *PRKDC *gene includes 86 exons (very cumbersome sequencing), and that the expression of DNA-PKcs protein, composed by 4096 aminoacids, can be normal but with defect of function [67].

HSCT is particularly problematic in all SCID caused by defects of V(D)J recombination, especially if radiosensitive (see later).

#### 2) Defects of signaling through the pre-T cell receptor

##### CD3δ, CD3ε, CD3ζ or CD3γ defect (OMIM 608971; 608971; 610163; 186830)

Complete CD3δ, CD3ε, CD3ζ or CD3γ defect can cause AR T^-^B^+^NK^+ ^SCID (about 1% of SCID): the different CD3 subunits, organized as *γε*, *δε *and ζζ dimers, join to pre-TCR and then TCR and are essential for their assemblage in cell membrane and signal transmission and therefore thymic T lymphopoiesis and mature T lymphocyte activation; defects cause differently severe phenotypes [68,69].

##### CD45 defect (OMIM 608971)

Very rare is also the AR T^-^B^+^NK^+/- ^SCID caused by CD45 defect [70]: CD45 (LCA, Leucocyte Common Antigen) is a protein-phosphotyrosine phosphatase essential for pre-TCR and TCR (and BCR also) signaling, constitutes about 10% of the proteins of T and B lymphocyte membrane, and exists in multiple isoforms produced by complex alternative splicing of the exons encoding its extracellular domains. The expression of the different CD45 isoforms depends on cell type and state of differentiation and activation (e.g., naïve CD4^+^*CD45RA^+ ^*T cells, and memory CD4^+^*CD45RO^+ ^*T cells); it is also very interesting the association between CD45 polymorphisms and susceptibility or resistance to infective or autoimmune diseases [71].

##### ZAP-70 defect (OMIM 176947)

ZAP-70 defect causes a characteristic deficiency of CD8^+ ^T lymphocytes, manifested as AR T^+ ^(CD4^+^CD8^-^) B^+^NK^+ ^SCID or as less severe phenotypes [16,72].

##### p56lck defect

A single case of AR T^-^B^+^NK^+ ^SCID caused by p56lck defect has been reported (not identified mutation, but aberrant splicing with exon 7 absence in the transcribed mRNA and very reduced protein expression) [73]; p56lck (OMIM 153390) defective activity has been also observed in adults with idiopathic CD4 lymphopenia [74].

### SCID caused by increased lymphocyte apoptosis

#### Reticular dysgenesis (RD) (OMIM 267500)

Reticular dysgenesis associates AR T^-^B^-^NK^- ^SCID and severe congenital neutropenia (arrest of myeloid maturation at the promyelocyte stage) with total leukocytes < 400/μL ("aleukocytosis"), fatal neonatal sepsis, no response to granulocyte colony-stimulating factor (G-CSF), and severe sensorineural deafness in babies surviving after successful HSCT. RD is caused by mutations (missense mutations; deletions) of the *AK2 *gene, encoding the mitochondrial enzyme adenylate kinase 2 (AK2) [75,76]. Adenylate kinases (AK) catalyze the reversible transfer of a phosphoryl group from adenosine triphosphate to adenosine monophosphate: 2 ADPs < == > ATP + AMP; from bacteria to humans, every cell survives only if ATP/ADP/AMP concentrations maintained within very narrow and tightly regulated ranges [77]. Seven different AK isozymes exist in human cells, with different tissue and subcellular distribution: AK1 (cytosol) and AK2-AK3-AK4 (mitochondrial) are present in all cells *except for blood nucleated cells and cells of stria vascularis in the inner ear, that express only AK2*. AK2 is located in the mitochondrial intermembrane space, the same as HAX-1 whose defect causes Severe Congenital Neutropenia of the families originally described by Kostmann [78].

#### Adenosine deaminase deficiency (ADA-SCID) (OMIM 102700)

The purine salvage enzyme adenosine deaminase (ADA) catalyzes the irreversible deamination of adenosine (Ado) and 2-deoxyadenosine (dAdo) to inosine and to deoxyinosine, respectively, and its deficiency results in "*metabolic poisoning*" from accumulation of Ado, dAdo and deoxyadenosine triphosphate (dATP). Excess intracellular dAdo and dATP cause generalized lymphocyte apoptosis (---> T^-^B^-^NK^- ^SCID), while excess extracellular Ado (normally produced by CD4^+ ^T_Reg _lymphocytes) acts on specific receptors with further lymphocyte inhibition [79] and might be important for frequent manifestations of immune dysregulation and autoimmunity (type I diabetes, hypothyroidism, autoimmune thrombocytopenia, hemolytic anemia) also reported in ADA-SCID patients treated by HSCT or gene therapy [80]. In humans the highest ADA enzyme activity is found in lymphocytes, particularly in intrathymic immature T cells, but ADA is an ubiquitous "housekeeping" enzyme present in all cell types; therefore, ADA deficiency is a 'systemic' metabolic disorder causing SCID as well as several nonimmunological abnormalities: alterations of the ribs (costochondral junctions), vertebral bodies, iliac crests and other skeletal segments [81]; neonatal hepatitis; renal and lung abnormalities; sensorineural deafness; neurological anomalies (cognitive, motor and behavioral problems) with a poor prognosis also after the correction of the immune defect [82]. IQ < -2 SD correlates with dATP levels at diagnosis; also note that the isolated genetic deficiency of the enzyme S-adenosyl homocysteine hydrolase (SAHH), inhibited by Ado, causes severe psychomotor delay [83].

Really, different therapeutic options exist for the treatment of ADA-SCID [84,85]:

- fully HLA-matched sibling or family donor HSCT without any conditioning is the treatment of choice, with success > 90%; at present, data from matched unrelated donor HSCT are not conclusive; by contrast, mismatched HSC transplants (parental haploidentical donor; mismatched unrelated donor), both without conditioning and with myeloablative or reduced-intensity conditioning, have poor chance of success and should be avoided;

- enzyme replacement therapy (ERT): weekly or twice-weekly intramuscular injection of PEG-ADA (polyethylene-glycol-modified calf intestinal ADA) protects from "metabolic poisoning" both lymphocytes (restoring immune function within 2 to 4 months) and other cells, and it is often life-saving therapy at diagnosis; ERT gives an overall 80% probability of surviving at 20 years; in the remaining 20% of patients, early mortality (within 6 months) results from serious conditions already present at diagnosis, late mortality from refractory hemolytic anemia, chronic respiratory insufficiency, lymphoproliferative disorders, liver malignancies; note that long-term (8-10 years) PEG-ADA treated patients show a gradual decline of thymic function and T cell counts;

- autologous HSC gene therapy (GT): as described recently [85,86], gene therapy had in ADA-SCID patients the most promising results.

#### Purine nucleoside phosphorylase (PNP) deficiency (OMIM 613179)

Purine nucleoside phosphorylase (PNP) follows ADA in the purine salvage pathway, and PNP deficiency also causes SCID (excess deoxyguanosine and deoxyguanosine triphosphate cause apoptosis of lymphocytes, mainly immature T lymphocytes). Note that PNP deficient cells do not produce uric acid, and therefore low uric acid in serum (< 2 mg/dL, usually < 1 mg/dL) supports the diagnosis. PNP deficiency has a poor prognosis [87]: PEG-PNP is not commercialized, and gene therapy is still experimental in mice; HSCT is the only therapy, but it does not correct the severe neurological problems usually present (hypertonia, hypotonia, ataxia, psychomotor delay); autoimmunity (hemolytic anemia, autoimmune thrombocytopenia, neutropenia, arthritis, etc.) and neoplasia are also frequent.

### SCID caused by defects in thymus embryogenesis

Thymocytes cannot develop to normal mature T lymphocytes without cross-talk with thymic cells (thymic epithelial cells, TECs; thymic stromal cells; thymic medullary dendritic cells) [88,89]; two forms of SCID recognize a primary embryonic thymic defect:

#### Nude/SCID Syndrome (OMIM 601705)

The gene *WHN *(wingled helix naked) encodes the FOXN1 (forkhead box N 1) transcription factor selectively expressed in thymic epithelia and skin, and its mutations cause the mouse Nude/SCID phenotype and also the equivalent human Nude/SCID syndrome (congenital total alopecia, and absence of the thymus with AR T^-^B^+^NK^+ ^SCID) [29,90]. The human form has been first identified in two sisters of a small Italian village, Acerno, where because of a founder effect the 6.52% of inhabitants are heterozygous carriers of the mutation exon 5 C792T R255X [91]. FOXN1 appears also essential for the embryonic development of the neural tube: an affected fetus, with absence of thymus and abnormal skin, showed also anencephaly and spina bifida; and, in the village there is a high rate of abortions in the first trimester in the marriages between heterozygous for the FOXN1 mutation [92].

#### Complete DiGeorge anomaly

Complete DiGeorge anomaly, characterized by absence of the thymus with consequent T^-^B^+^NK^+ ^SCID and variously associated facial dismorphy, congenital heart disease (conotruncal malformations), and neonatal hypocalcemia by defect of parathyroid glands, has different etiologies [93]:

- approximately 50%: **DiGeorge Syndrome **(OMIM 188400) [94] by 22q11.2 deletion of about 3 Mb interesting > 35 genes, among which the *TBX-1 *(T-box 1) gene involved in the development of heart, thymus, parathyroid glands, palate, face; note that the vast majority of infants with DiGeorge syndrome have "partial" DiGeorge anomaly, with low T cell counts but not the immunodeficiency of complete DiGeorge anomaly;

- about 25%: **CHARGE association **(coloboma, heart defects, atresia choanae, retardated growth and development, genital hypoplasia, ear anomalies/deafness; OMIM 214800), with in the majority of patients mutation of the *CHD7 *gene, encoding the chromodomain helicase DNA-binding protein-7 [95,96];

- about 15%: **diabetic mother embryopathy **[97].

22q11.2 deletion and CHD7 mutation have autosomal dominant transmission, with de novo mutation in more than 80% of cases; in around 1/3 of the cases, SCID manifests as Omenn syndrome ("atypical" complete DiGeorge anomaly).

These children have *normal hematopoietic stem cells *that cannot develop to mature T lymphocytes because of the absence of thymic stroma and epithelium; so, the specific treatment of thymic aplasia is *thymus transplant*: neonatal thymus took during newborn heart surgery and cultured in vitro to eliminate the HLA not-matched thymocytes, and then insertion of multiple slides containing thymic epithelium (that is, surprisingly, functionally not HLA-restricted) into the quadriceps muscle of the SCID infant [98]. However, thymus transplant is a technically very demanding procedure, and there is only one center in the United States (Duke University Medical Center, Durham, NC 27710, USA) that is currently able to accomplish it [99]. Alternatively, a satisfactory T cell immunity may also be obtained in these children by the *adoptive transfer of HLA identical expanding and long-lasting T lymphocytes *present in simple peripheral blood mononuclear cells (PBMC), bone marrow, or cord blood of a fully HLA-matched donor [100].

### SCID caused by impaired calcium flux

#### ORAI1 defect-STIM defect

The variations of intracellular Ca^2+ ^levels are a fundamental mechanism for the signal transduction in all living cells [101]. In T lymphocytes, the activation of the TCR/CD3 complex causes a release of intracellular Ca^2+ ^from the endoplasmic reticulum (ER) stores, followed by a "store operated Ca^2+ ^entry" (SOCE) that is a conspicuous influx into the cell of other Ca^2+ ^ions from extracellular space because of opening of the Ca^2+ ^release-activated calcium channels (CRAC) of the cell membrane; a main final effect of intracellular Ca^2+ ^increase is translocation of NFAT (nuclear factor of activated T cells) into the nucleus, with activation of specific genes.

In rare AR T^+^B^+^NK^+ ^SCID patients has been identified a SOCE-CRAC defect, caused by mutations in genes encoding two highly conserved proteins: ORAI1 (subunit forming pores in CRAC; its name comes from ORAI, the three sisters of Greek mythology), and STIM1 (stromal interaction molecule-1; it is the sensor of Ca^2+ ^levels in ER and the activator of ORAI1-CRAC).

The clinical phenotypes of the ORAI1 defect (OMIM 612782) and of the STIM defect (OMIM 612783) are similar [15]: serious AR T^+^B^+^NK^+ ^SCID, with normal T, B and NK cell differentiation and counts but severe T lymphocyte defect of Ca^2+ ^influx and of proliferation after mitogen stimulation, *ipergammaglobulinemia *but with deficiency of specific antibody production, congenital non-progressive global myopathy (hypotonic infant), ectodermal dysplasia (anhydrosis, defects in the formation of dental enamel). In the STIM1 defect there is also CD4^+ ^T_Reg _lymphocyte deficiency, with severe autoimmunity (autoimmune thrombocytopenia, hemolytic anemia), enlarged lymph nodes and epatosplenomegaly.

### SCID caused by other mechanisms

#### Coronin-1A defect

Mice with a homozygous missense mutation in the Coronin-1A (*CORO1A*) gene show severe T cell lymphopenia; this has suggested the recent discovery of the absence of Coronin-1A in a 13-month-old girl with AR T^-^B^+^NK^+ ^SCID, by deletion of the entire *CORO1A *gene on one allele (600 kbs deletion in 16p11.2) and a dinucleotide deletion resulting in frameshift and premature termination on the other allele [102,103]. Coronins (1A, 1B, 1C, 2A, 2B, 7) are a highly conserved family of proteins, regulators of cell F-actin structures, cytoskeletal rearrangements and intracellular membrane transport; they, by contrast to WASP (Wiskott-Aldrich Syndrome Protein) antagonize actin polymerization. Coronin-1A (OMIM 605000) is especially expressed in T cells, and its defect causes an excess of F-actinin in the cortex thymocytes with drastically impaired cell movement, intrathymic retention of single positive CD4^+^CD8^- ^or CD4^-^CD8^+ ^mature T lymphocytes, and severe peripheral T lymphopenia [104].

#### MHC Class II (MHCII) defect (OMIM 209920)

The genes *CIITA*, *RFXANK*, *RFX5, RFXAP *encode four factors that regulate promoters and transcription of the HLA DR, DP, DQ cluster, localized at 6p21.3; their mutations cause absence of Major histocompatibility complex class II (MHCII) molecules, normally expressed at cell surface by thymic epithelial cells, activated T lymphocytes, and cells (B lymphocytes, dendritic cells, monocytes/macrophages) that present antigens to CD4^+ ^T lymphocytes. Apart from a minority of "attenuated" cases, typical MHC class II deficiency causes a serious AR T^+ ^(CD4 ^-^CD8^+^) B^+^NK^+ ^SCID with a poor prognosis and overall cure rate < 50% also by HSCT from familial HLA-matched donor. HSCT in these patients is complicated by a high incidence of acute GvHD associated with preexisting viral infections, and it is highly recommended that HSCT be performed in young children (< 2 years), using either an HLA-identical sibling or the best available compatible donor [105].

#### CHH (Cartilage hair hypoplasia) (OMIM 250250)

Mutations of the highly polymorphic *RMRP *(ribonuclease mitochondrial RNA processing) gene, encoding not a protein but the 267-nucleotide-long ***RNA ***component of the mitochondrial RNA-processing endonuclease (a multiprotein RNA complex with at least ten different proteins) cause defects in ribosomal RNA processing and mitochondrial and cellular replication, and a heterogeneous phenotypic spectrum [37]. Cartilage hair hypoplasia, particularly frequent in the Amish and the Finnish populations (respectively 1:19 and 1:76 individuals carriers of the mutation g.70 A > G, because of a founder effect) is a metaphyseal chondrodysplasia with short-limbed dwarfism, light-colored hypoplastic hair, and variable immunodeficiency: AR T^-^B^+^NK^+ ^SCID (also manifested as Omenn syndrome), selective CD8^+ ^T lymphopenia, or even not relevant immunologic defects; patients with combined immunodeficiency without skeletal alterations have also been reported. Apart from CHH, mutations of *RMRP *(usually in different nucleotides of the RNA molecule) cause other three skeletal disorders: metaphyseal dysplasia without hypotrichosis (MDWH), kyphomelic dysplasia, and anauxetic dysplasia.

#### Hoyeraal-Hreidarsson Syndrome (HHS) (OMIM 300240)

**Figure 8:**
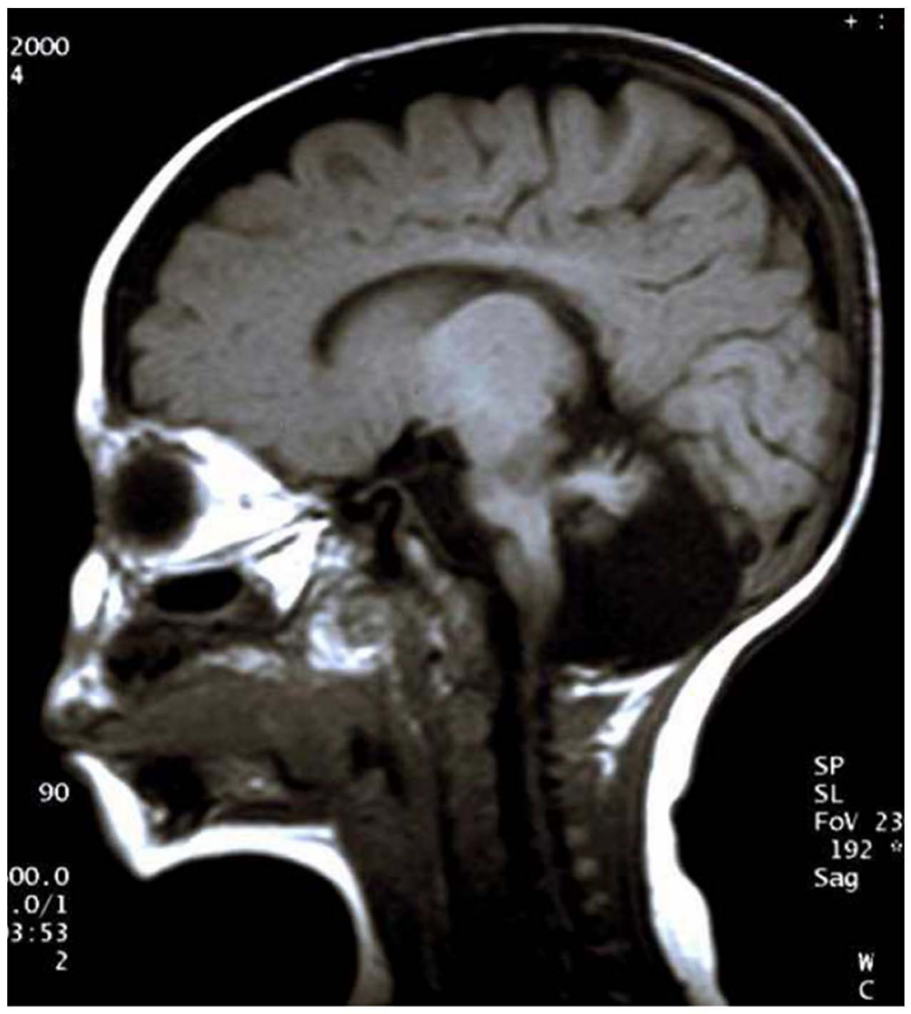
**Hoyeraal-Hreidarsson Syndrome**. Cerebellar hypoplasia in a Sardinian 6-month-old male with T^+^B^-^NK^- ^SCID; missense mutation of *DKC1 *gene encoding dyskerin (X-linked Hoyeraal-Hreidarsson syndrome).

Hoyeraal-Hreidarsson syndrome (HHS), characterized by telomerase defect and by the pathognomonic association of T^+^B^-^NK^- ^SCID and *cerebellar hypoplasia *(Figure 8), is the severe infantile variant of dyskeratosis congenita [17,106]. Defective telomerase activity affects all tissues in constant renewal (bone marrow, skin, oral and gut epithelium, lung alveolar epithelium, etc.) and also development, differentiation and activation of lymphocytes; it also causes extreme toxicity by pre-HSCT conditioning (especially by alkylating agents and by irradiation), similar to defects of NHEJ.

XL-HHS and the "classical" X-linked dyskeratosis congenita (XL-DC) affect only males and are due to mutations of the *DKC1 *gene within Xq28, encoding dyskerin, a component of telomerase [17]. Various other genetic defects cause HHS both in male and female infants: AR-HHS by homozygous mutations of the *TERT *gene encoding telomerase reverse-transcriptase [107]; AD-HHS by heterozygous mutations of the *TINF2 *gene encoding TIN2 (TRF1-interacting nuclear protein 2), one of the components (six proteins) of the shelterin telomere protection complex [108,109]; and AD-HHS by heterozygous mutation of *DCLRE1B *(DNA cross-link repair protein 1B) gene, encoding Apollo, a DNA repair factor interacting with the shelterin complex (mutation recently identified by Revy *et al*. in the HHS female infant described by them ten years ago) [110,111].

#### Hereditary folate malabsorption (HFM) (OMIM 229050)

Hereditary folate malabsorption (HFM) is due to proton-coupled folate transporter (*PCFT*) gene mutations and consequent folate malabsorption in the duodenum and upper jejunum, with depletion of transplacentally acquired stores at 3-4 months of age; PCFT also transports folate through choroid plexus into the cerebrospinal fluid. Lymphocytes and developing nervous system are particularly sensitive to the defective thymidine synthesis and resulting DNA instability caused by folate deficiency. Affected infants show failure to thrive, diarrhea, anemia (megaloblastic, but masked to normocytic if associated iron deficiency), infections, hypogammaglobulinemia and an AR T^+^B^+^NK^+ ^SCID phenotype; they also present seizures and, if not treated promptly, severe neurodevelopmental defects. Serum and cerebrospinal fluid folate levels are undetectable.

This is a special reversible SCID: parenteral folate repletion causes a dramatic recovery of immunodeficiency and, if prompt and aggressive, prevents irreversible neurological damages [112].

## Hematopoietic stem cell transplantation (HSCT)

SCID can be successfully treated by HSCT, the only curative option for most affected infants; several recent papers [113-116] report on survival and long-term outcome in several hundreds of patients over the last decades, with a success rate ranging from 70-95 percent and varying incidence of adverse effects and complications.

Main variables influence the outcome of HSCT [117]:

• **Age and clinical condition at the time of diagnosis and of HSCT: **there is an always increasing evidence of best outcome if diagnosis and HSCT performed in the first few months of life (1-4 months), before clinical presentation with infections and failure to thrive; of course, this underlines the importance of population-based newborn screening for SCID, that permits the early diagnosis of asymptomatic newborns.

• **HSC donor: **unfortunately, only a minority of SCID infants have an HLA-matched sibling, obviously the ideal donor; for infants without a matched related donor, alternative donors are an HLA-matched unrelated volunteer or an HLA-matched unrelated banked cord blood unit, or, if none of these is available in a very short time, rigorously T-depleted haploidentical related (mother or father) bone marrow or peripheral blood stem cells (PBSC).

• **No conditioning versus conditioning regimen, and SCID genotype/phenotype: **HSCT engraftment without any pretransplant conditioning regimen is theoretically possible in most SCID infants, especially if NK^- ^SCID (e.g., ADA-SCID, common γc defect, JAK3 defect), but with frequently defective donor B cell reconstitution and so persistent B cell deficiency that requires long-term (for the rest of life) immunoglobulin replacement therapy. New, now in use, cytoreductive nonmyeloablative regimens, particularly those including fludarabine, appear safe and causing a reduced toxicity and presumably reduced long-term effects on for example growth and endocrine system: therefore they are probably recommended, if possible, also in all these very young (few months of age) children [118].

In SCID infants with V(D)J recombination - DNA repair defects (especially NHEJ defects) the molecular problem causes cellular radiosensitivity and extreme toxicity by "historical" pretransplant conditioning regimen (especially by alkylating agents and, obviously, by irradiation); the same is true in patients with telomerase defect (Hoyeraal-Hreidarsson syndrome and also its "later" form, classical dyskeratosis congenita).

Not only because of the different sensitivity to conditioning regimen, SCID genotype/phenotype influence the outcome of HSCT: e.g., a better outcome is usual in SCID with common γc or JAK3 defect (a part from frequent cutaneous HPV) and IL-7Rα defect; on the contrary, a complicated outcome is more frequent in SCID with V(D)J recombination - DNA repair defects (high risk of conditioning toxicity, obviously if not "disease-specific" strategy for nonmyeloablative regimen; and, also if normal immunologic reconstitution, frequent autoimmunity, gut problems, etc.) and in ADA-SCID (frequent neurological anomalies: cognitive, motor and behaviour problems) with a poor prognosis also after the correction of the immune defect.

## Gene Therapy (GT)

Gene therapy, i.e., the insertion of normal gene through a vector virus (e.g., defective Moloney murine leukemia virus, M-MLV) into DNA of autologous CD34^+ ^hematopoietic stem cells, progenitors also of lymphocytes, had practical application in two forms of SCID with different results [50,86,119,120].

• Beginning in 1999 in Paris and in 2001 in London two European groups performed gene therapy for treating **common γc defect XL-SCID **in respectively 11 and 10 children, aged from 1 to 33 months; 17 of 21 children had satisfactory T cell reconstitution, and 12 of them had also normal B cell reconstitution with no more need for immunoglobulin replacement therapy. Unfortunately, 5 children (4 in Paris, 1 in London) at 24-68 months from gene therapy developed an acute lymphoblastic leukemia (ALL) mainly because complex mutagenesis started with the insertion at 11p13 of a single copy of the defective retrovirus, containing the normal *IL2RG *gene but also its enhancer, near the promoter of *LMO2 *(LIM domain only 2), known oncogene of T lymphocytes then aberrantly transcribed and expressed (**insertional mutagenesis**) [121,122]. One child was dead from leukemia, the others were cured by chemotherapy; the occurrence of this serious complication led to stop of gene therapy for common γc defect XL-SCID in Europe. In the United States a gene therapy trial, now closed for new patients, recluted 8 patients already treated unsuccessfully with allogeneic HSCT: in the first 3 patients (aged 10-14 years), immunological recovery has been poor, probably due to age-related thymic involution and chronic viral infections.

• Gene therapy had very good results in **ADA-SCID **children, and it is really indicated in ADA-SCID patients without an HLA-matched related donor, as particularly described in the 15 patients (age at GT 6 months - 5.6 years) of the casistic of Aiuti *et al*. [86]. Aiuti used an ameliorated transduction protocol of CD34^+ ^autologous stem cells by a defective MLV vector, nonmyeloablative conditioning (busulfan 2 mg/kg ev at days -3 and -2) to create space in the bone marrow, and stopping of PEG-ADA treatment to give a selective proliferative vantage to lymphocytes with corrected gene. All 15 patients are alive, with in the first 10 treated patients: good immunological recovery in 9/10, persistent expression of ADA with "systemic detoxification" without any more need for PEG-ADA until 8 years since gene therapy in 8/10, and no more need for immunoglobulin replacement therapy in 5/10. Similar results have been reported in patients treated by other groups. Especially, in no patient has been reported the complication of the clonal leukemic proliferation because of insertional mutagenesis, and this also if the viral vector however inserts itself dangerously in proximity of LMO2 or other known oncogenes.

There is now an intense research for different viral vectors that do not present problems of insertional mutagenesis: SIN (self-inactivating) Lentiviruses integrate also in the cells not in mitosis, and (on the contrary of gammaretrovirus such as MLV) do not have as preferential integration site the promoter regions of active (expressed) genes [123]. The recent report of a clonal expansion from integration of Lentivirus vector in the DNA-binding protein HMGA2 (high mobility group AT-hook 2) gene in a patient of the gene therapy trial for thalassemia in France [124] imposes however further caution.

## Conclusions

More than thirty identified genetic defects cause human SCID and certainly novel genes and molecular mechanisms will be discovered over the next few years, maybe between function-based candidate genes (e.g., *PTCRA *gene encoding the invariant pre-TCRα chain; genes encoding scaffold proteins involved in immune signaling; genes encoding DNA repair proteins).

Human SCID is always a prenatal disorder of T lymphocyte development: it is already present at birth even if clinically silent in most affected newborns, therefore the universal newborn screening for SCID (as started in Wisconsin) is really of great practical importance. If not identified at birth, SCID manifests itself in the first few months of life as typical lymphopenic T^- ^SCID or atypical T^+^/T^++ ^SCID (especially Omenn syndrome), but always as a pediatric emergency. Essentially every affected infant could be cured by very early diagnosis, prompt prevention (sterile rooms!) and treatment of infections, and timely hematopoietic stem cell transplantation (or, if ADA deficiency, gene therapy or enzyme replacement therapy). Knowledge of the different genetic and clinical forms of SCID is essential for the most accurate approach to diagnosis and treatment as well to family counseling.

## Consent

Written informed consent was obtained from the parents of the infants for publication of images and clinical data in Figure 3, Figure 4 and Figure 8. A copy of the written consent is available for review by the Editor-in-Chief of this journal.

## Competing interests

The author declares that he has no competing interests.

## Author's information

Pediatric HSCT Unit, 2^ Pediatric Clinic of University, Ospedale Microcitemico, Via Jenner s/n, 09121 Cagliari, Sardinia, Italy.
